# Role of RANKL (TNFSF11)-Dependent Osteopetrosis in the Dental Phenotype of *Msx2* Null Mutant Mice

**DOI:** 10.1371/journal.pone.0080054

**Published:** 2013-11-21

**Authors:** Beatriz Castaneda, Yohann Simon, Didier Ferbus, Benoit Robert, Julie Chesneau, Christopher Mueller, Ariane Berdal, Frédéric Lézot

**Affiliations:** 1 INSERM, UMR 872, Centre de Recherche des Cordeliers, Laboratoire de Physiopathologie Orale Moléculaire, Equipe 5, Paris, F-75006 France; Université Paris-5, Paris, F-75006; Université Paris-6, Paris, F-75006 France; Université Paris-7, Paris, F-75006 France; 2 Department of Basic Studies, Faculty of Odontology, University of Antioquia, Medellin, Colombia; 3 CNRS, URA 2578, Institut Pasteur, Unité de Génétique Moléculaire de la Morphogenèse, Paris, F-75015 France; 4 INSERM UMR 957, Equipe Ligue Contre le Cancer 2012, Nantes, F-44035 France; 5 Université de Nantes, Laboratoire de Physiopathologie de la Résorption Osseuse et Thérapie des Tumeurs Osseuses primitives, EA3822, Nantes, F-44035 France; 6 CNRS, UPR 9021, Institut de Biologie Moléculaire et Cellulaire (IBMC), Laboratoire Immunologie et Chimie Thérapeutiques, Université de Strasbourg, Strasbourg, F-67084 France; Team ‘Evo-Devo of Vertebrate Dentition’, France

## Abstract

The MSX2 homeoprotein is implicated in all aspects of craniofacial skeletal development. During postnatal growth, MSX2 is expressed in all cells involved in mineralized tissue formation and plays a role in their differentiation and function. *Msx2* null (*Msx2* ^−/−^) mice display complex craniofacial skeleton abnormalities with bone and tooth defects. A moderate form osteopetrotic phenotype is observed, along with decreased expression of RANKL (TNFSF11), the main osteoclast-differentiating factor. In order to elucidate the role of such an osteopetrosis in the *Msx2* ^−/−^ mouse dental phenotype, a bone resorption rescue was performed by mating *Msx2* ^−/−^ mice with a transgenic mouse line overexpressing *Rank (Tnfrsf11a)*. *Msx2* ^−/−^ *Rank^Tg^* mice had significant improvement in the molar phenotype, while incisor epithelium defects were exacerbated in the enamel area, with formation of massive osteolytic tumors. Although compensation for RANKL loss of function could have potential as a therapy for osteopetrosis, but in *Msx2* ^−/−^ mice, this approach via RANK overexpression in monocyte-derived lineages, amplified latent epithelial tumor development in the peculiar continuously growing incisor.

## Introduction

Mutations in muscle segment homeobox (MSX) transcription factors cause craniofacial malformations such as cleft palate for MSX1 and craniosynostosis (Boston type) for MSX2 [1]. An MSX2 mutation is associated with amelogenesis imperfecta [2], highlighting the importance of this protein in dental epithelial cell differentiation and function. The fact that MSX2 is required for normal dental epithelial cell fates is supported by the *Msx2* ^−/−^ mouse dental phenotype. These mutant mice present amelogenesis imperfecta and root dysmorphia associated with differentiation defects in epithelial cells (i.e., ameloblastic tumors and defects in Hertwig epithelial root sheaths [HERS] and epithelial cell rests of Malassez) [3]–[5]. In addition, *Msx2* ^−/−^ mice display dentinogenesis imperfecta and regional and graded osteopetrosis from the first to the third molar, with inclusion of the mandibular third molar [3]. Molar inclusion can give rise to tooth ankylosis and odontogenic tumor formation [3]–[4], as described for other osteopetrosis mouse models (*Src* and ntl mutants) [6]–[7]. However, the role of osteopetrosis in the multifaceted dental phenotypes observed in *Msx2* ^−/−^ mice is unclear. Previous studies have suggested that osteopetrosis in *Msx2* ^−/−^ mutants could have two nonexclusive origins. First, because *Msx2* is expressed during growth by a subpopulation of alveolar bone osteoclasts [3], this osteoclast subset may be missing in the null mutants. Second, gene expression of the key osteoclast differentiation factor RANKL is severely decreased in the dental epithelium and alveolar bone of *Msx2* ^−/−^ mice [3]–[5]. Therefore, in order to investigate the importance of osteopetrosis in *Msx2* ^−/−^ mouse dental defects, we developed a strategy to rescue bone resorption by overexpressing RANK in osteoclast precursors [8] of *Msx2* ^−/−^ mice. The phenotypes of different teeth were then analyzed in these mice.

## Materials and Methods

### Animal generation and sampling

Ethics statement: the Consultative Bioethics Committee for Health and Life Science has specifically approved the present study (CEEA-2011-32). Staff trained to perform in vivo studies did all of the experiments.

*Msx2* knockout (KO) mice were generated by replacing the entire coding sequence of *Msx2* with the bacterial *LacZ* gene [3]. *Rank* transgenic mice were generated by heterologous recombination of a cassette containing 3.2 kb of the human myeloid related protein 8 (MRP8, also known as S100A8) gene promoter and the coding DNA sequence of the mouse *Rank* gene. Approximately 30 copies were inserted in tandem in the transgenic line [8].

Males that were heterozygous for the *Msx2* gene mutation and overexpressed *Rank* were mated with females heterozygous for the *Msx2* gene mutation in order to generate all possible *Msx2* and *Rank* genotypes. The genetic background of all of the mice was CD1 Swiss. Mice were studied at 2, 3, 4, 8, 10, and 16 weeks, with at least three animals in each experimental group for a total of 147 animals.

### Microradiographs, histological analyses, tartrate-resistant acid phosphatase (TRAP) activity assays, and keratin 14 immunohistochemistry

After anesthesia of the mice, intracardiac perfusions were performed with a fixative solution containing 4% paraformaldehyde (Sigma, la Verpillière, France) in phosphate-buffered saline (PBS) pH 7.4. Complete fixation was ensured by immersion of the heads in fixative solution overnight at 4°C. After rinsing in PBS, the head halves (cut along the sagittal axis) were microradiographed on High Resolution Film SO-343 (Kodak Professional, Paris, France) with a microfocal X-ray generator (Tubix, Paris, France) at a focal distance of 56 cm for 20 min (power setting: 12 mA and 15 kV). The head halves were then processed for histology by decalcification at 4°C for up to 2 months (depending on the age of the samples) in a pH 7.4 PBS solution that contained 4% EDTA (Sigma) and 0.2% paraformaldehyde. After extensive washing in PBS, the samples were dehydrated in increasing concentrations of ethanol and toluene and were finally embedded in paraffin (Paraplast plus, Sigma). Serial frontal sections of the head halves were sliced with a microtome (RM 2145; Leica, Rueil-Malmaison, France). The 7-µm-thick sections were deparaffinized and rehydrated before being either stained according to a modified van Gieson protocol [9], assayed for tartrate-resistant acid phosphatase (TRAP) activity as previously described [9], or immunolabeled for cytokeratin-14. Briefly, after saturation for 1 h with 10% horse serum in 1×PBS, sections were incubated overnight at 4°C with anti-keratin-14 rabbit primary antibody (PRB-155P; Covance, Paris, France). After washing in 1×PBS, an anti-rabbit biotinylated secondary antibody (BA-1100; Vector Laboratories, Burlingame, CA, USA) was applied for 1 h. Sections were then washed, treated with streptavidin–alkaline phosphatase conjugate (Roche, Meylan, France), and stained with nitro-blue tetrazolium and 5-bromo-4-chloro-3′-indolyphosphate (NBT/BCIP, Roche).

### Micro-computed tomography scanner imaging

A micro-CT scanner (desktop Skyscan 1172; Skyscan, Aartselaar, Belgium) was used to provide three-dimensional images of mouse mandibles. This system is based on a cone-beam X-ray source. A spatial resolution that produced voxels that measure 6.7 µm per side was used. Acquisition parameters were 80 kV anode voltage and 100 mA for an exposition time of 4 s. A 0.25° rotation step was performed between two expositions. A total of five expositions were obtained for each angle, and means were calculated. For each mode, a 0.5-mm aluminum filter was installed in the beam path to block the softest X-rays and to increase the accuracy of the beam-hardening correction (BHC). Cross-sectional images were reconstructed with a classical Feldkamp cone-beam algorithm with NRecon (Skyscan). Three-dimensional reconstructions were achieved with the software package CTAn (Skyscan). A threshold between 40 and 140 was selected, because it provided the best image of the mandible and suppressed artifacts.

### RT-PCR and TaqMan array RT-qPCR analyses

Dissections of 2-week-old mouse mandibles (five mice per group) were performed under a stereomicroscope in order to collect alveolar bones and incisor epithelia, as previously described [5]. Tissues were directly immersed in RNA extraction solution (Tri-Reagent; Euromedex, Souffelweyersheim, France), and the extraction was performed according to the manufacturer's instructions. For classical RT-PCR, reverse transcription was performed on 1 µg of total RNA with Superscript II (Gibco, Cergy-Pontoise, France) and hexanucleotide random primers (Gibco), and PCRs were done with Eurobiotaq (Eurobio, Courtaboeuf, France), following the manufacturer's instructions. The following sets of primers chosen in different exons were used: RankTg-Fw ATG TCT CTT GTC AGC TGT CTT; RankTg-Rv GCT CAT AAT GCC TCT CCT G; Rank-Fw CTT GGA CAC CTG GAA TGA AGA AG; Rank-Rv AGG GCC TTG CCT GCA TC; Rankl-Fw CAG CAT CGC TCT GTT CCT GT; Rankl-Rv TCG TGC TCC CTC CTT TCA TC; Opg-Fw TGA TGA GTG TGT GTA TTG CAG C; Opg-Rv CCC AGG CAA ACT GTC CAC CAA; Runx2-Fw GGA CGA GGC AAG AGT TTC AC; Runx2-Rv TGC CTG CCT GGG ATC TGT AA; Ocn-Fw CTC ACT CTG CTG GCC CTG; Ocn-Rv CCG TAG ATG CGT TTG TAG GC; CD3e-Fw ACT GGA GCA AGA ATA GGA; CD3e-Rv AGG AGA GGA AAG GAA CTG; CD19-Fw CCA TCG AGA GGC ACG TGA A; CD19-Rv TCC ATC CAC CAG TTC TCA ACA G; F4/80-Fw AGA TGG GGG ATG ACC ACA CTT C; F4/80-Rv TGT TCA GGG CAA ACG TCT CG; Csf1r-Fw GAC TTC GCC CTC AGC TTG G; Csf1r-Rv TCC CCA GAC CCC TCA TGT T; CD11b-Fw TGG GCA GGT GGA GCC TTC CT; CD11b-Rv CAC TGC CAC CGT GCC CTC TG; CD11c-Fw CTG AGA GCC CAG ACG AAG ACA; CD11c-Rv TGA GCT GCC CAC GAT AAG AG; 18S-Fw AAA CGG CTA CCA CAT CCA AG; 18S-Rv CCT CCA ATG GAT CCT CGT TTA. PCR products were separated by 2% agarose gel electrophoresis and were photographed with a Bio-Rad Gel Doc XR camera (Bio-Rad, Marnes-la-Coquette, France).

For TaqMan quantitative RT-PCR arrays, reverse transcription was performed on 1 µg of total RNA with the High Capacity cDNA reverse transcription kit (Applied Biosystems, Foster City, CA, USA) and PCR was performed with a 7900HT Fast system real-time PCR apparatus using Taqman mouse immune arrays (Applied Biosystems) according to the manufacturer's instructions.

### Statistical analyses

Data were analyzed using a one-factor analysis of variance to assess the effects of genotype. As appropriate, post-hoc testing was performed using Fisher's Protected Least Significant Difference (PLSD). Differences were considered significant at *p*≤0.05. Data are presented as means ± standard error of the mean (S.E.M).

## Results and Discussion

**Figure 1:**
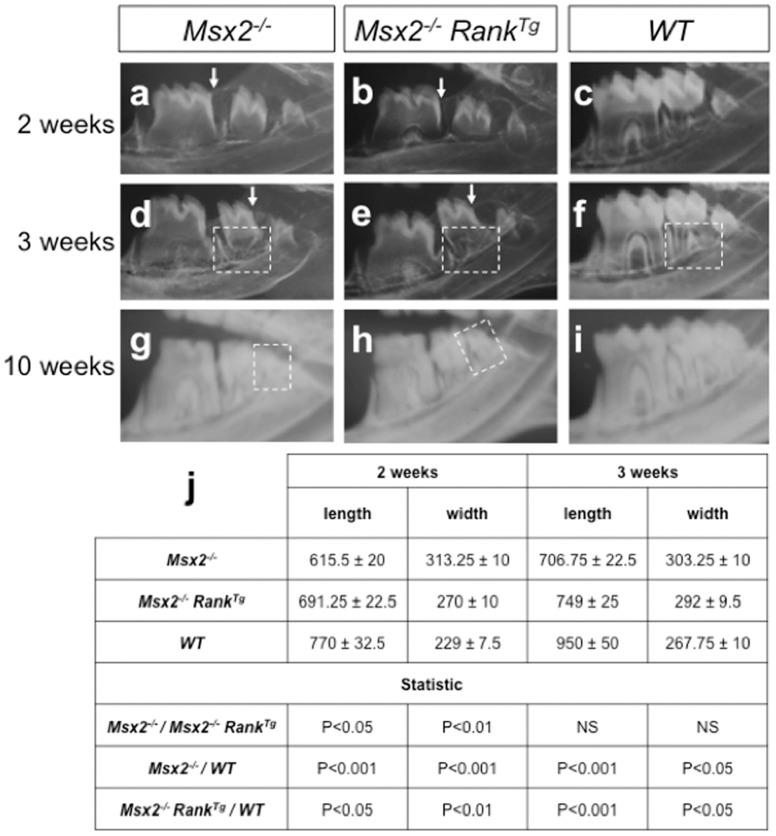
Effect of transgenic *Rank* on lower molar growth in *Msx2* ^−^^/−^ mice. Microradiographs were taken at 2 (a–c), 3 (d–f), and 10 (g–i) weeks for *Msx2* ^−/−^ mice overexpressing (b, e, and h) or not expressing (a, d, and g) transgenic *Rank*, and for *WT* mice (c, f, and i). At 2 and 3 weeks, eruption of the first and second molars was more advanced in *Msx2* ^−/−^ *Rank^Tg^* mice than in *Msx2* ^−/−^ mice, as shown by their positions relative to the vestibular bone crest (arrows in a, b, d, and e). At 3 weeks, the most significant feature of the progression in second molar growth was the more advanced root elongation in *Msx2* ^−/−^ *Rank^Tg^* mice compared to *Msx2* ^−/−^ mice (squares in e versus d). However, the root lengths did not match those of *WT* mice (square in f). At 10 weeks, while the third molars of *Msx2* ^−/−^ mice were completely surrounded by and indistinguishable from bone on the microradiograph (square in g), the third molars of *Msx2* ^−/−^ *Rank^Tg^* mice were fully erupted and functional (square in h). Measures of the first molar mesial root length and width at the median position (in µm) were performed on histological sections and presented in a table form (j). A higher length and lower width were observed at 2 weeks for *Msx2* ^−/−^ *Rank^Tg^* molar root comparatively to *Msx2* ^−/−^ molar root. However, *Msx2* ^−/−^ *Rank^Tg^* molar root length and width remained respectively lower and superior to those observed for WT molar root. At 3 weeks, no significant difference of length and width was observed between *Msx2* ^−/−^ *Rank^Tg^* and *Msx2* ^−/−^ molar roots.

**Figure 2:**
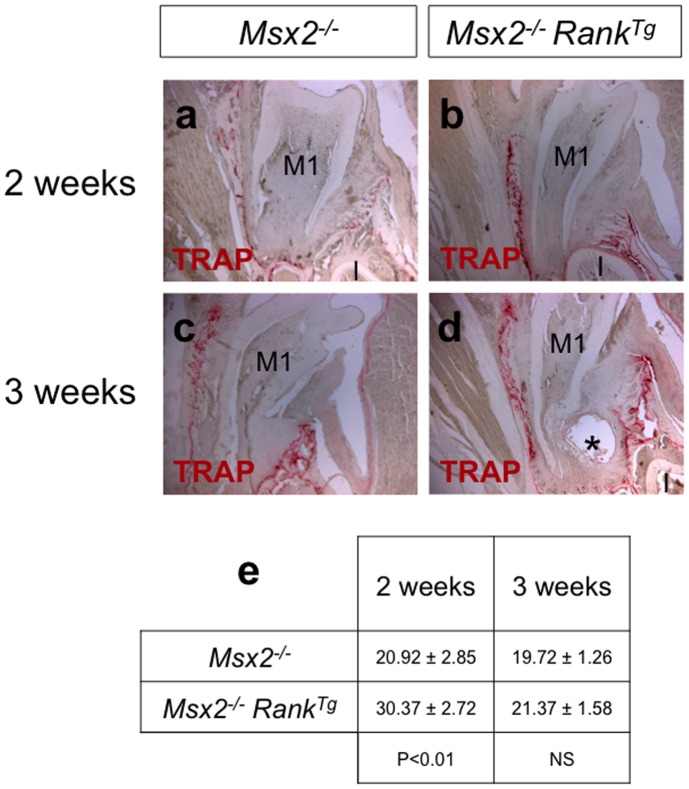
*Rank* overexpression stimulates alveolar bone osteoclastogenesis. TRAP activity assays were performed on frontal sections of the mandibles of 2 (a, b) and 3 (c–f) week-old mice to determine the effect of *Rank* overexpression on osteoclast numbers. At 2 weeks, the number of TRAP-positive cells was significantly increased around the first molar root in *Msx2* ^−/−^ *Rank^Tg^* mice (b, e). The root appeared longer but thinner in *Msx2* ^−/−^ *Rank^Tg^* than in *Msx2* ^−/−^ mice, and advanced eruption was also clearly visible. At 3 weeks, no significant difference in the number of TRAP-expressing cells was observed (c, d, e). While the length of the first molar roots of *Msx2* ^−/−^ mutants expressing or not expressing *Rank* was similar, it remained thinner in *Msx2* ^−/−^ *Rank^Tg^* mice (c, d). Asterisk in (d): Epithelial cyst on the lingual part of the root of a *Msx2* ^−/−^ *Rank^Tg^* mouse. M1, first molar; I, incisor. (e) Numbering of the TRAP positive cells in the alveolar bone surface performed on 7 µm thick sections (n>8) and presented as a table with statistical analyses.

Analyses of *Msx2* ^−/−^ mouse molars revealed delayed tooth eruption and shortened roots (Fig. 1a, d, g) [3], [9]. RANK overexpression on an *Msx2* ^−/−^ background (*Msx2* ^−/−^ *Rank^Tg^*) resulted in significant recovery of all molar eruption and root elongation processes, as revealed by the relative positions of the teeth and alveolar bone crests (arrows in Fig. 1a–e), full eruption of the third molar (square in Fig. 1g–h), and the greater length of the molar roots at day 14 comparatively to *Msx2* ^−/−^ mouse molar (square in Fig. 1d–f; Fig. 2a, b; Fig. S4c). Measures of *Msx2* ^−/−^, *Msx2* ^−/−^ *Rank^Tg^* and WT mouse mandible first molar mesial root length and width at 2 and 3 weeks, performed on histological sections (three animals by group) using Image-J software, confirmed that, at 2 weeks, roots are significantly longer (p<0.05) in *Msx2* ^−/−^ *Rank^Tg^* molars comparatively to *Msx2* ^−/−^ molars but remained shorter (p<0.05) than WT molars (Fig. 1J). Moreover, the root width was significantly reduced in *Msx2* ^−/−^ *Rank^Tg^* molars comparatively to *Msx2* ^−/−^ molars (p<0.01) as previously described [9] but was superior (p<0.01) to WT molars (Fig. 1J).

At 3 weeks, mandible first molar mesial root length and width were not significantly different (p>0.5) in *Msx2* ^−/−^ *Rank^Tg^* and *Msx2* ^−/−^ mice (Fig. 1j) but were respectively significantly lower (p<0.001) and higher (P<0.05) than WT mouse ones (Fig. 1j).

**Figure 3:**
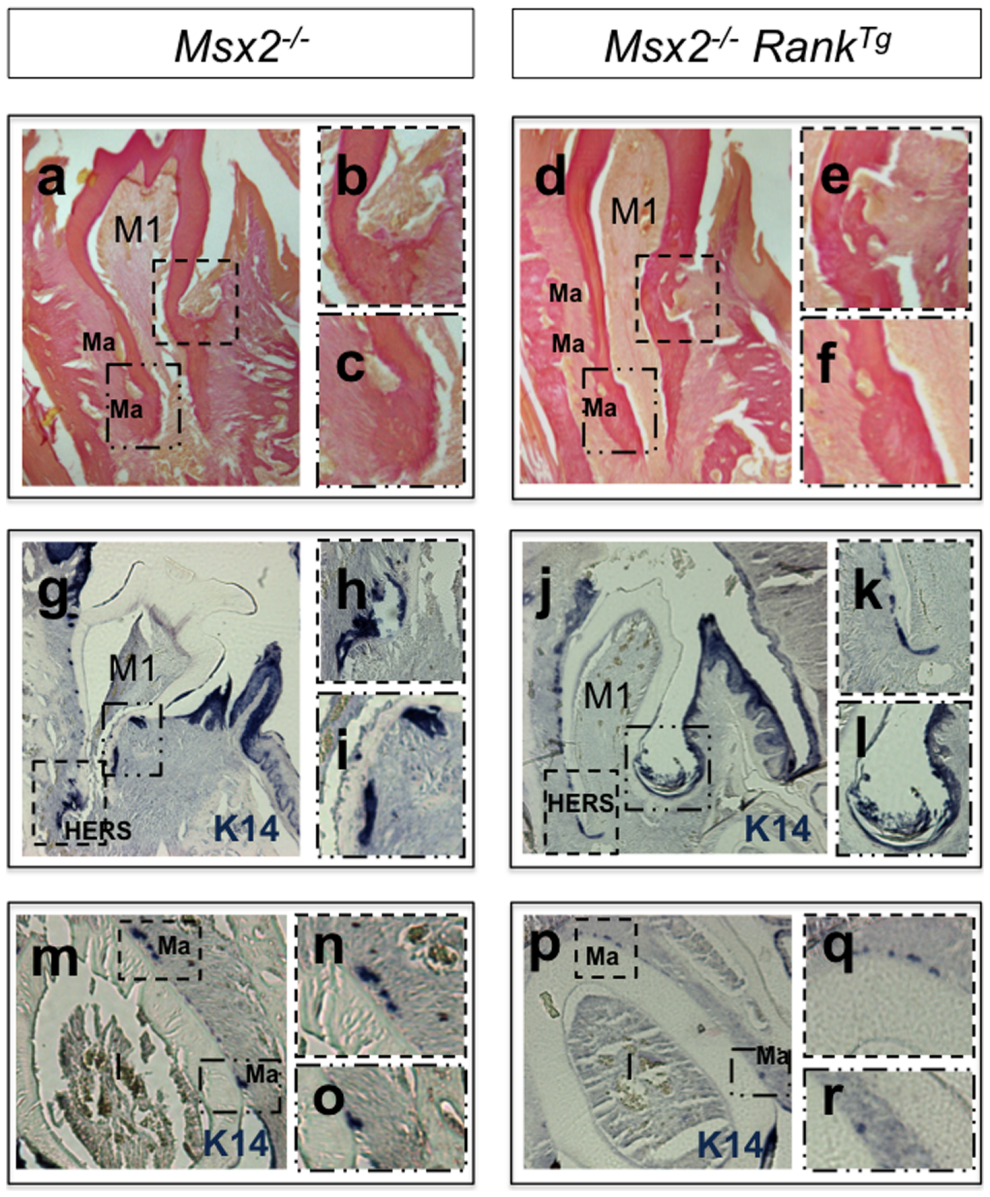
Effect of transgenic *Rank* on lower first molar and incisor root formation in *Msx2* ^−^^/−^ mice. Van Gieson histology staining (a–f) and keratin immunohistochemistry (g–r) were respectively performed on mandibular frontal sections of 3- and 2-week-old *Msx2* ^−/−^ mice either overexpressing or not expressing transgenic *Rank*. At 3 weeks, *Rank* overexpression had induced a normalization in the size of most epithelial cell rests of Malassez (Ma) (a and c versus d and f), and at 2 weeks it had induced a better commitment of Hertwig epithelial root sheath (HERS) cells, specifically in the labial area (j and k versus g and h). Occasionally and independently of *Rank* overexpression, epithelial cyst–like structures were observed in the lingual area of *Msx2* ^−/−^ mandibular first molars (j, i). Cytokeratin-14 immunolabelling revealed that these cyst-like structures were associated with abnormal continuity between dental and oral epithelia (j) and the presence of a periodontal pocket (square in j enlarged in l). Another defect observed at 3 weeks in the lingual root of *Msx2* ^−/−^ mice, also independent of *Rank* overexpression, was a lacuna-like structure in the dentine facing the site of transition between crown and root epithelia (squares in a and d enlarged in b and e, respectively). In the incisor root equivalent, normalization of the size of the epithelial cell rests of Malassez (Ma) was observed in *Msx2* ^−/−^ *Rank^Tg^* mice (squares in m versus p enlarged in n and o and q and r, respectively).

RANK overexpression resulted in a significant (p = 0,0014) increased osteoclast numbers at 2 weeks (Fig. 2), a better commitment of HERS cells in the labial area (Fig. 3g, h, j, k), and a normalization in the size (volume measured using Image-J software) of most of the epithelial cell rests of Malassez (Fig. 3a, c, d, f; Fig. S1). However, the root morphology of *Msx2* ^−/−^ mice was not completely restored. The roots remained shorter than in wild-type (*WT*) mice (Fig. 1g–j) [9]. Moreover, epithelial cyst-like structures that were occasionally observed in the lingual area of the mandibular first molar mesial root in *Msx2* ^−/−^ mice (Fig. S2a) were also present in the *Msx2* ^−/−^ *Rank^Tg^* mutants (asterisk in Fig. 2d), at an approximately similar frequency, suggesting that the origin of these cyst-like structures was associated with MSX2 loss of function in epithelial cells. Keratin-14 immunostaining showed that these structures were associated with apparent continuity between dental and oral epithelia (Fig. 3j) and the formation of a periodontal pocket (square in Fig. 3j enlarged in 3l). Interestingly, cyst-like structures were only observed in the lingual part of the root. This asymmetrical localization may be associated with a labial-lingual gradient of transcription and growth factor expression during tooth morphogenesis and initial histogenesis [10]–[11]. Indeed, MSX2 loss may affect the expression or function of other factors; for example, DLX2 is known to be a key MSX2 partner [12].

Another defect observed in the lingual root of *Msx2* ^−/−^ mice, independent of RANK overexpression, was the presence of a lacuna-like structure in the dentine at the crown-root transition site (squares in Fig. 3a, d enlarged in 3b, e, respectively). These structures were maintained in the adult (asterisks in Fig. S2b, c).

**Figure 4:**
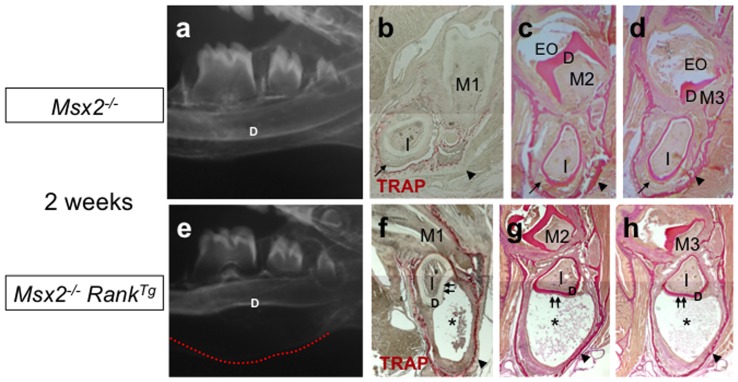
Effect of *Rank* overexpression on lower incisors of *Msx2* ^−^^/−^ mice. Mandibular microradiographs (a, e) and TRAP activity assays (b, f) or van Gieson histology staining (c, d, g, h) of mandibular frontal sections were performed to characterize the effect of *Rank* overexpression on the lower incisors of 2-week-old *Msx2* ^−/−^ mice. Substantial enlargement of the area between the basal bone and the dentin was observed in *Msx2* ^−/−^ *Rank^Tg^* mice (e) compared to *Msx2* ^−/−^ mice not expressing *Rank^Tg^* (a). This enlarged area, which corresponds to the incisor epithelial compartment, was associated with abnormal curvature in both basal bone (red dotted line) and dentin (D). Mandibular frontal sections through the first (M1), second (M2), and third (M3) molar planes revealed that the enlargement corresponds to an epithelial hypertrophy with the presence of an internal necrosis-like area (asterisks in f–h). In *Msx2* ^−/−^ incisors, no hypertrophy of the epithelium was visible, but this tissue was disorganized and lacked the ameloblastic palisade structure (arrows in b–d). There was also a substantial increase in the number of osteoclasts around the incisors of *Msx2* ^−/−^ *Rank^Tg^* mice (f) compared to *Msx2* ^−/−^ mice not expressing *Rank^Tg^* (b). Moreover, the thickness of the mandibular basal bone in the *Msx2* ^−/−^ *Rank^Tg^* mutants appeared highly reduced compared to *Msx2* ^−/−^ mice not expressing *Rank^Tg^* (arrowheads in f–h versus b–d).

**Figure 5:**
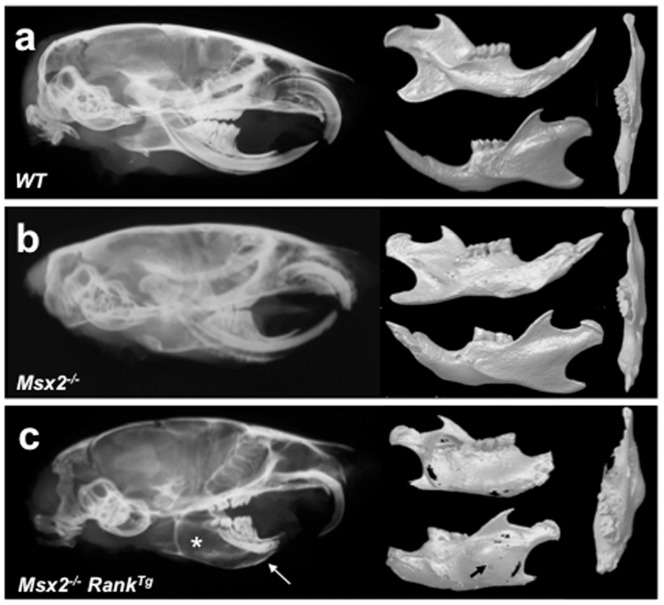
Combined effects of loss of *Msx2* and *Rank* overexpression on mouse mandibular bone phenotype. Microradiographs and scans of 16-week-old *WT* (a), *Msx2* ^−/−^ (b), and *Msx2* ^−/−^ *Rank^Tg^* (c) mouse skulls were performed to compare characteristics of the bone of the mandible. While the *Msx2* ^−/−^ mouse mandibular features (b) presented no major alterations compared to WT animals (a), *Msx2* ^−/−^ *Rank^Tg^* mice had marked disruptions in the architecture of the mandibular bone (c). These disruptions were either mono- or bilateral and were associated with conversion of the incisor epithelium toward massive osteolytic tumors (asterisks in c). Basal bone around these tumors was thinner (arrows in c), porous (arrow in c), and displaced, as seen in the upper view of the mandibular scan (c).

Similar to the molars, the defect in the root analog region of the incisors was improved by RANK overexpression, as reflected by a better commitment of HERS cells and the more typical size of the epithelial cell rests of Malassez (Fig. 3m–r). Strikingly, however, by 2 weeks, in the crown equivalent area of all incisors, the dental epithelium had converted into a massive osteolytic tumor (Fig. 4a–h). The tumor caused a deformation in the dentin (double arrows in Fig. 4f–h) and was associated with substantial resorption of the surrounding bone, as shown by the increased osteoclast numbers (Fig. 4b, f). The tumor caused total destruction of the mandible within 4 months (Fig. 5c). The increased osteoclasts around the incisor seemed to have a positive impact on tumor growth. This scenario is reminiscent of a previously described amplification loop between tumor cells and osteoclasts, which may occur in bone metastasis of several tumor types [13].

**Figure 6:**
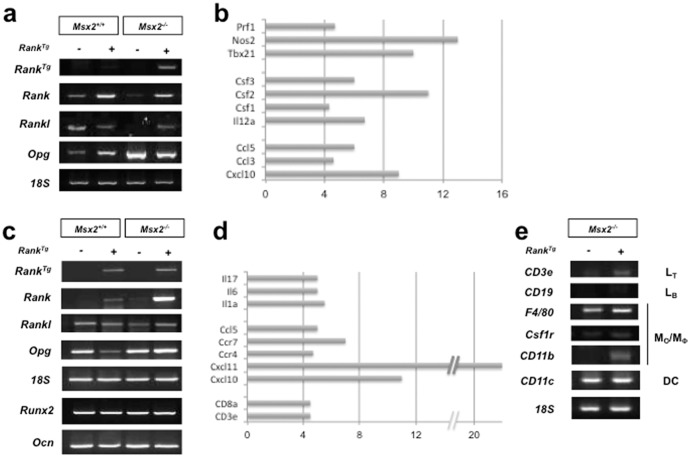
Gene signature induced by *Rank* overexpression in the dental epithelium and alveolar bone of *Msx2* ^−^^/−^ mice. RT-PCR of total RNA extracted from dental epithelium (a) and alveolar bone (c, e) revealed *Rank* expression in the epithelium and alveolar bone of *Msx2* ^−/−^ *Rank^Tg^* mice. Increased *Rankl* expression was associated with *Rank* overexpression in *Msx2* ^−/−^ mice (a, c). In *Msx2* ^−/−^ *mice*, *Rank* overexpression induced a decrease in *Opg* transcriptional activity in the epithelium, while in alveolar bone, expression of *Opg* increased slightly (a, c). Expression levels of *Runx2* and *Ocn* in alveolar bone were unaffected by *Rank* overexpression (c), but expression of the T lymphocyte marker *Cd3e* and the monocyte and macrophage markers *Csf1r*, *F4/80* and *Cd11b* were increased (e). Also shown are 4-fold or higher increases in gene expression in *Msx2* ^−/−^ *Rank^Tg^* mice compared to *Msx2* ^−/−^ mice not expressing *Rank^Tg^*, as quantified by RT-qPCR TaqMan arrays in dental epithelium (b) and alveolar bone (d).

The MSX2 homeoprotein is a critical factor for epithelial cell commitment in various organs, including skin and skin appendages [3], [14]. MSX2 misexpression was reported in tumors of these epithelial tissues in the context of bone metastasis [15] and osteolysis [16]. During bone resorption, MSX2 may positively regulate RANKL expression, as suggested by a reduction in *Rankl* expression in the dental epithelium of *Msx2* ^−/−^ mice (Fig. 6a) [3]–[5] and similar expression in odontogenic tumors [17]. This regulation is of particular importance, because increased RANKL expression in tumor cells is directly correlated with hyperactive bone resorption [18]. To further elucidate how RANK overexpression promotes the conversion of *Msx2* ^−/−^ mouse incisor epithelium into massive tumors, expression levels of *Rankl, Opg* (*Tnfrsf11b*), *Rank*, and various inflammation markers were comparatively analyzed in the epithelium of 14-day-old *WT* and *Msx2* ^−/−^ mice that lacked or expressed the *Rank* transgene (Fig. 6a, b). *Rankl*, *Rank*, and *Opg* expression were detected in *WT* mouse incisor epithelium. In contrast, in *Msx2* ^−/−^ mouse incisor epithelium, *Rankl and Rank* expression decreased but *Opg* expression increased (Fig. 6a), in accordance with the previously described osteopetrotic phenotype [3]. In *Rank^Tg^* mouse incisor epithelium, *Rank* and *Opg* expression was increased and *Rankl* expression decreased compared to *WT* epithelium (Fig. 6a), as previously reported [9]. These variations are explained by the more advanced stage of tooth eruption [9]. In the *Msx2* ^−/−^ *Rank^Tg^* mouse epithelium, *Rankl* and *Rank* expression was increased and *Opg* expression decreased compared to *Msx2* ^−/−^ mouse epithelium (Fig. 6a). These variations are consistent with the observed augmentation in the surrounding alveolar bone resorption at 2 weeks (Fig. 2a, b). Interestingly, transgene expression was observed only in *Msx2* ^−/−^ *Rank^Tg^* mouse epithelium (Fig. 6a), suggesting that cells within the tumor mass were expressing the transgene; these cells might correspond to monocyte-derived cells. To further characterize the immune cells infiltrating the epithelial tumor, TaqMan inflammation/cancer array analyses were performed. Elevated signals for *Prf1*, *Nos2*, and *Tbx21* (Fig. 6b; Fig. S3) are indicative of intra-tumoral T helper type 1 cytotoxic cells, in addition to monocyte-derived cells that are likely recruited and maintained by CSF1 (Fig. 6b). Cytotoxic cells may also be natural killer (NK) cells, which would correspond with the observed unaltered *CD8a* expression levels (Fig. S3) and increased transcription of genes encoding factors such as IL12A and CXCL10 (Fig. 6b; Fig. S3), which are known to stimulate NK cell chemotaxis and differentiation [19]. In response, NK cells produce IFNG, TNF, CSF2, CCL3, and CCL5 [19], which are all up-regulated in *Msx2* ^−/−^ *Rank^Tg^* mouse epithelium (Fig. 6b; Fig. S3). Further studies will be necessary to unravel the mechanistic relationship between inflammatory cell recruitment and epithelial tumor activation, and the relationship between tumor growth and RANKL expression. Keeping in mind that cells of the monocyte/macrophage lineage were present in the epithelial tumor (Fig. 6a), the recent finding that monocytes control NK cell differentiation in the context of antitumor immunity [20] constitutes an interesting basis for future studies.

The epithelial tumor growth resulted in marked resorption of the surrounding alveolar bone, likely due to increased osteoclast numbers (Fig. 4f). Interestingly, *Runx2* and osteocalcin transcripts remained stable, indicating unaltered bone apposition (Fig. 6c). The rise in osteoclast numbers is likely the result of the marked increase in *Rankl* expression (Fig. 6c). Analysis of different immune cell–lineage markers (Fig. 6e) suggested that cells of the myeloid linage were increased in *Msx2* ^−/−^ *Rank^Tg^* mouse alveolar bone. There also appeared to be an increase in the cytotoxic T lymphocyte population, as suggested by increased *CD3e* and *CD8a* expression (Fig. 6d, e). These data and the high expression levels of *Il1a*, *Il6*, *Il17 Ccl5*, *Ccr4*, and *Ccr7* (Fig. 6d) provide evidence for enhanced bone loss through inflammation, as described in other pathologies [21]. CXCL10 functions as a chemokine for monocytes and is implicated in osteoclastogenesis [22]–[25], with possible crosstalk with RANKL [24]. Thus, CXCL10 production may constitute a key element in the massive osteolytic epithelial tumor development observed in *Msx2* ^−/−^ *Rank^Tg^* mice by fostering an amplification cycle between tumor growth and alveolar bone resorption. CXCL11 was shown to inhibit osteoclastogenesis by a mechanism independent of its CXCR3 receptor [26]. CXCL11 should therefore interfere with increased bone resorption and tumor growth. On the other hand, CXCL11 is also known to activate T lymphocytes [27], which could amplify inflammation of the bone environment and adjacent epithelia, where increased *Cxcr3* transcriptional activity was observed (Fig. S3).

In addition to above described effects of RANK over-expression on *Msx2* ^−/−^ dental phenotype, benefic effects of such over-expression have also been observed in other skeleton sites known to be affect in *Msx2* ^−/−^ mouse (Fig. S4). For instance, the characteristic open foramen of *Msx2* ^−/−^ mouse skull was partly closed in RANK over-expressing mutant (Fig. S4a). Similarly, the *Msx2* ^−/−^ mouse tibia that presented features of soft osteopetrosis switched to rather osteopenic bone in RANK over-expressing mutant (Fig. S4b). Nevertheless, other skeleton defects associated to MSX2 lost were poorly improved by RANK over-expression as the tibia length that remained shorter than WT mouse one (Fig. S4b).

## Conclusion

In conclusion, rescuing bone resorption in *Msx2* ^−/−^ mice by overexpressing RANK in the osteoclastic lineage allowed for the correction of a substantial portion of the molar abnormalities, most likely by counteracting the decrease in RANKL expression, which is correlated with *Msx2* ^−/−^ osteopetrosis. From a more general viewpoint, our results indicate that functional compensation may be a promising approach for the treatment of osteopetrosis. However, in this mouse model, in which *Msx2* was not expressed and RANK was overexpressed, and which features continuously growing incisors, precocious formation of a massive and osteolytic odontogenic epithelial tumor was observed.

## Supporting Information

Figure S1 Comparative analysis of epithelial rest of Malassez sizes in roots of wild type, *Msx2^−/−^* and *Msx2^−/−^ Rank^Tg^* mice. Whatever the age considered, the RANK over-expression in the *Msx2^−/−^* mouse normalized the size of the rest of Malassez. Measures were realized as previously described [5] using Image-J software.
(TIF)

Figure S2 Van Gieson staining of *Msx2^−/−^* mouse mandible first molar frontal sections at 3, 4 and 16 weeks.The presence of a cyst-like structure at the root lingual surface was observed at 3 weeks (a). At 4 and 16 weeks lacunae in the dentin area facing the site of transition between crown and root epithelium was present (asterisk in b–c). D: dentine; PDL: periodontal ligament; P: pulp.
(TIF)

Figure S3 Table of the TaqMan immune arrays results. Numbers corresponded to the induction folds observed in the 14 day-old *Msx2^−/−^ RANK^Tg^* mouse tissues comparatively to *Msx2^−/−^* mouse tissues. Negative numbers corresponded to reduction folds and ND means that no expression difference has been detected. Inductions over 4 folds have been reported in Figure 6.
(DOCX)

Figure S4 Combined effects of loss of *Msx2* and *Rank* overexpression on mouse skull, tibia and molar phenotypes. Upper and lateral scan-views of skulls of 14 day-old wild type, *RANK^Tg^*, *Msx2^−/−^* and *Msx2^−/−^ RANK^Tg^* mice (a) enabled to see a significant reduction of the open foramen in the *Msx2^−/−^ RANK^Tg^* mouse comparatively to *Msx2^−/−^* mouse while sutures were normal in *RANK^Tg^* mouse as in *WT* mouse. Scan-sections along tibias of 14 day-old *WT*, *RANK^Tg^*, *Msx2^−/−^* and *Msx2^−/−^ RANK^Tg^* mice (b) evidenced that RANK over-expression moderately increased *Msx2^−/−^* mouse tibia length without reached the normal size seen in *RANK^Tg^* and *WT* mice. BV/TV measures on sections realized at 50, 30 and 15% of tibias length evidenced that RANK over-expression is able to reverse the mild osteopetrosis phenotype seen in *Msx2^−/−^* mouse toward a rather osteopenic phenotype also visible in *RANK^Tg^* mouse. Lateral scan-views of mandible first molars of 14 day-old wild type, *RANK^Tg^*, *Msx2^−/−^* and *Msx2^−/−^ RANK^Tg^* mice (c) enabled to see a significant reduction of the mesial root length in *Msx2^−/−^* and *Msx2^−/−^ RANK^Tg^* mice comparatively to WT mouse with however a longer root in *Msx2^−/−^ RANK^Tg^* mouse comparatively to *Msx2^−/−^* mouse.
(TIF)
