# Supplementary figures and images for: Role of RANKL (TNFSF11)-Dependent Osteopetrosis in the Dental Phenotype of Msx2 Null Mutant Mice
Source: PLoS One. 2013 Nov 21;8(11):e80054. doi: 10.1371/journal.pone.0080054 (PMC3836916; doi:10.1371/journal.pone.0080054)

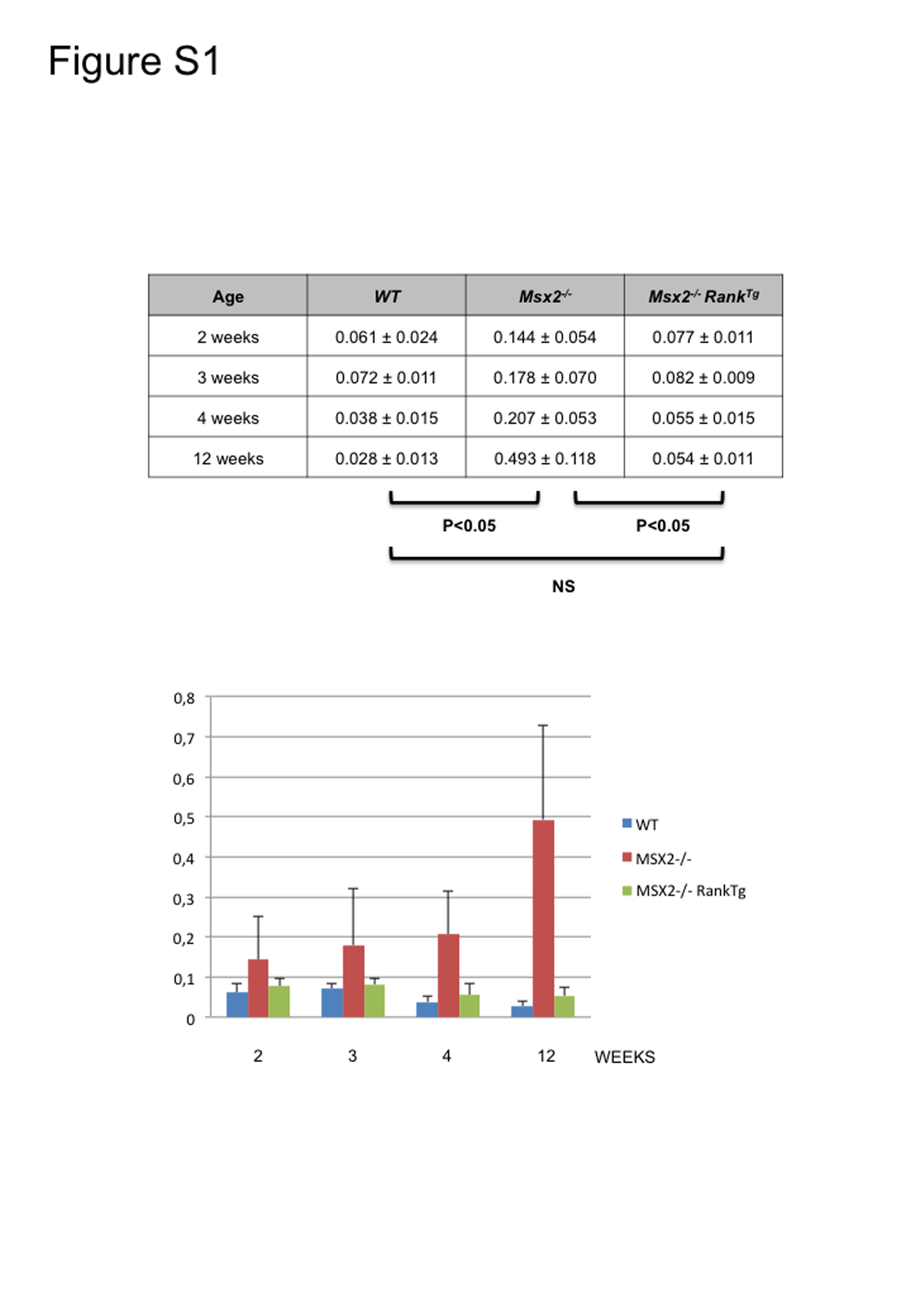

Supplement: Figure S1 — Comparative analysis of epithelial rest of Malassez sizes in roots of wild type, Msx2−/− and Msx2−/− RankTg mice. Whatever the age considered, the RANK over-expression in the Msx2−/− mouse normalized the size of the rest of Malassez. Measures were realized as previously described [5] using Image-J software. (TIF) [file pone.0080054.s001.tif]

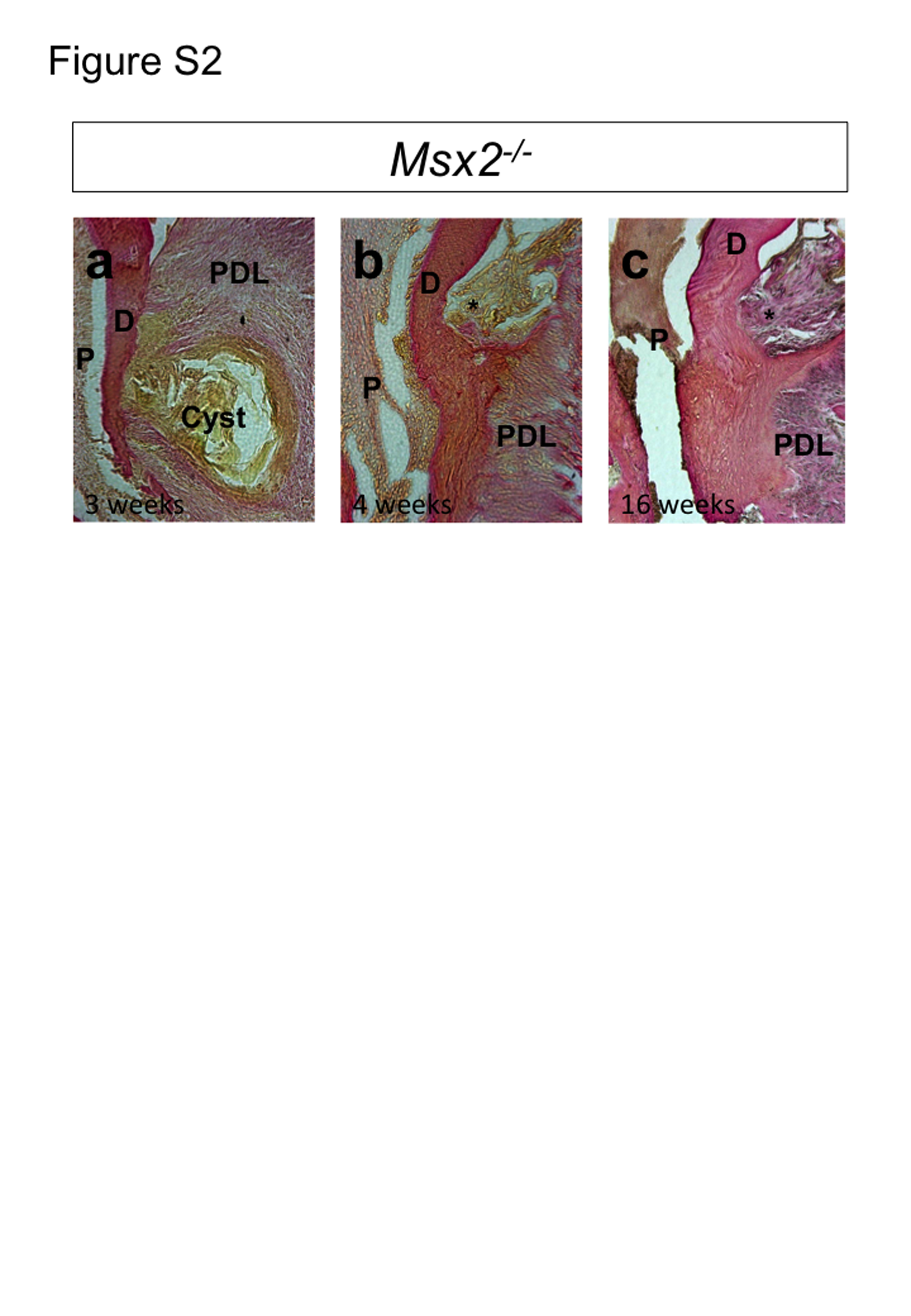

Supplement: Figure S2 — Van Gieson staining of Msx2−/− mouse mandible first molar frontal sections at 3, 4 and 16 weeks.The presence of a cyst-like structure at the root lingual surface was observed at 3 weeks (a). At 4 and 16 weeks lacunae in the dentin area facing the site of transition between crown and root epithelium was present (asterisk in b–c). D: dentine; PDL: periodontal ligament; P: pulp. (TIF) [file pone.0080054.s002.tif]

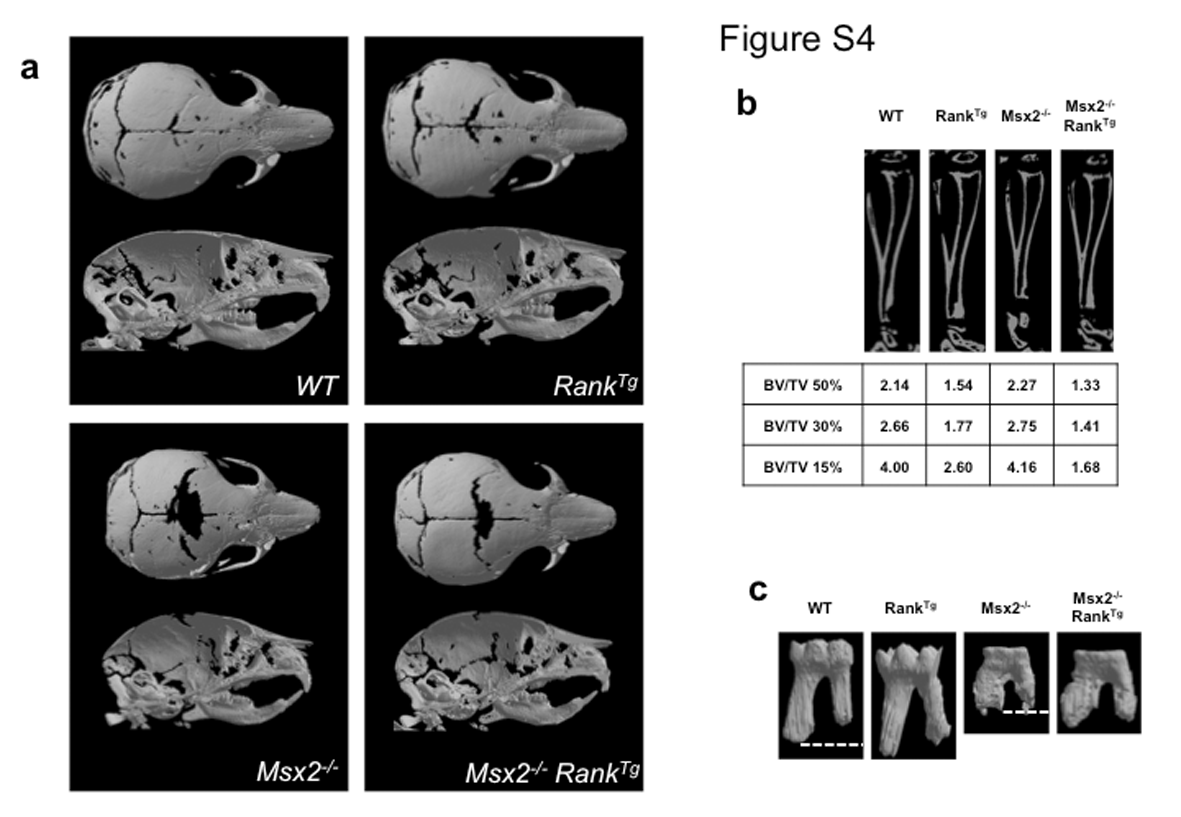

Supplement: Figure S4 — Combined effects of loss of Msx2 and Rank overexpression on mouse skull, tibia and molar phenotypes. Upper and lateral scan-views of skulls of 14 day-old wild type, RANKTg, Msx2−/− and Msx2−/− RANKTg mice (a) enabled to see a significant reduction of the open foramen in the Msx2−/− RANKTg mouse comparatively to Msx2−/− mouse while sutures were normal in RANKTg mouse as in WT mouse. Scan-sections along tibias of 14 day-old WT, RANKTg, Msx2−/− and Msx2−/− RANKTg mice (b) evidenced that RANK over-expression moderately increased Msx2−/− mouse tibia length without reached the normal size seen in RANKTg and WT mice. BV/TV measures on sections realized at 50, 30 and 15% of tibias length evidenced that RANK over-expression is able to reverse the mild osteopetrosis phenotype seen in Msx2−/− mouse toward a rather osteopenic phenotype also visible in RANKTg mouse. Lateral scan-views of mandible first molars of 14 day-old wild type, RANKTg, Msx2−/− and Msx2−/− RANKTg mice (c) enabled to see a significant reduction of the mesial root length in Msx2−/− and Msx2−/− RANKTg mice comparatively to WT mouse with however a longer root in Msx2−/− RANKTg mouse comparatively to Msx2−/− mouse. (TIF) [file pone.0080054.s004.tif]
