# Supplementary material for: Role of RANKL (TNFSF11)-Dependent Osteopetrosis in the Dental Phenotype of Msx2 Null Mutant Mice
Source: PLoS One. 2013 Nov 21;8(11):e80054. doi: 10.1371/journal.pone.0080054 (PMC3836916; doi:10.1371/journal.pone.0080054)
Supplement: Figure S3 — Table of the TaqMan immune arrays results. Numbers corresponded to the induction folds observed in the 14 day-old Msx2−/− RANKTg mouse tissues comparatively to Msx2−/− mouse tissues. Negative numbers corresponded to reduction folds and ND means that no expression difference has been detected. Inductions over 4 folds have been reported in Figure 6. (DOCX) [file pone.0080054.s003.docx]

Figure S3

| Symbol | Gene name | Alveolar bone | Epithelium |
| --- | --- | --- | --- |
| Agtr2 | Angiotensin II Receptor Type 2 | ND | ND |
| Bax | Bcl2 Associated X protein | ND | ND |
| Bcl2 | B-cell cll/lymphoma 2 | ND | ND |
| Bcl2l1 | Bcl2 related protein, long isoform, included | ND | 2.5 |
| C3 | Complement Component 3 | 2 | ND |
| Ccl19 | Chemokine CC motif, Ligand 19 | ND | ND |
| Ccl2 | Chemokine CC motif, Ligand 2 | 2,5 | ND |
| Ccl3 | Chemokine CC motif, ligand 3 | ND | 4.6 |
| Ccl5 | Chemokine CC motif, ligand 5 | 5 | 6 |
| Ccr2 | Chemokine CC motif Receptor 2 | ND | ND |
| Ccr4 | Chemokine CC motif Receptor 4 | 4.7 | -2.8 |
| Ccr7 | Chemokine CC motif Receptor 7 | 7 | ND |
| Cd19 | CD19 Antigen | -4 | ND |
| Cd28 | CD28 Antigen | 2 | ND |
| Cd34 | CD34 Antigen | ND | ND |
| Cd38 | CD38 Antigen | ND | 2.3 |
| Cd3e | CD3 Antigen Epsilon Subunit | 4.5 | ND |
| Cd4 | CD4 Antigen | ND | ND |
| Cd68 | CD68 Antigen | ND | 3 |
| Cd80 | CD80 Antigen | 2.7 | 1.5 |
| Cd86 | CD86 Antigen | ND | ND |
| Cd8a | CD8 Antigen alpha polypeptide | 4.5 | ND |
| Col4a5 | Collagen type IV alpha5 | ND | ND |
| Csf1 | Colony stimulating factor 1 | ND | 4.3 |
| Csf2 | Colony stimulating factor 2 | ND | 11 |
| Csf3 | Colony stimulating factor 3 | ND | 6 |
| Ctla4 | Cytotoxic T Lymphocyte associated 4 | ND | ND |
| Cxcl10 | Chemokine cxc motif ligand 10 | 11 | 9 |
| Cxcl11 | Chemokine cxc motif ligand 11 | 22 | 3.3 |
| Cxcr3 | Chemokine cc motif receptor 3 | ND | 4 |
| Cyp1a2 | Cytochrome P450 Subfamily 1A, Polypeptide 2 | ND | ND |
| Cyp7a1 | Cytochrome P450 Subfamily VIIA, Polypeptide 1 | ND | ND |
| Ece1 | Endothelin-converting enzyme 1 | ND | ND |
| Edn1 | Endothelin 1 | ND | ND |
| Fas | Tumor Necrosis Factor Receptor Superfamily member 6 | ND | ND |
| Fn1 | Fibronectin 1 | ND | ND |
| Gusb | Beta glucuronidase | ND | ND |
| Gzmb | Granzyme B | 2 | 4 |
| H2-Ea | Histocompatibility 2, class II antigen E beta | ND | -14 |
| H2-Eb1 | Histocompatibility 2 class II antigen B beta | 4 | ND |
| Hmox1 | Heme Oxigenase 1 | ND | ND |
| Icos | Inducible T cell Costimulator | 2 | ND |
| Ifng | Interferon gamma | ND | 3 |
| Ikbkb | Inhibitor of Kappa Light chain gene enhancer in B-cells, Kinase Beta | ND | ND |
| Il10 | Interleukin 10 | 2.4 | 4 |
| Il12a | Interleukin 12alpha | ND | 6.7 |
| Il12b | Interleukin 12beta | ND | -8 |
| Il13 | Interleukin 13 | ND | ND |
| Il15 | Interleukin 15 | ND | ND |
| Il17 | Interleukin 17 | 5 | ND |
| Il18 | Interleukin 18 | ND | ND |
| Il1a | Interleukin 1alpha | 5.5 | 3 |
| Il1b | Interleukin 1beta | 2 | ND |
| Il2 | Interleukin 2 | ND | ND |
| Il2ra | Interleukin 2 receptor alpha | ND | ND |
| Il3 | Interleukin 3 | ND | ND |
| Il4 | Interleukin 4 | ND | ND |
| Il5 | Interleukin 5 | ND | ND |
| Il6 | Interleukin 6 | 5 | 3 |
| Il7 | Interleukin 7 | ND | 2 |
| Il9 | Interleukin 9 | ND | ND |
| Lrp2 | Low density lipoprotein receptor-related protein 2 | ND | ND |
| Lta | Lymphotoxin alpha | ND | ND |
| Nfkb1 | Nuclear factor kappa beta subunit 1 | ND | ND |
| Nfkb2 | Nuclear factor kappa beta subunit 1 | ND | ND |
| Nos2 | Nitric oxide synthase 2A | 2.5 | 13 |
| Prf1 | Perforin 1 | ND | 4.7 |
| Ptgs2 | Prostaglandin endoperoxide synthase 2 | ND | 3.7 |
| Ptprc | Protein-tyrosine phosphatase receptor type C | ND | ND |
| Sele | Selectin e | ND | 2 |
| Selp | Selectin P | ND | ND |
| Ski | V-ski avian sarcoma viral oncogene homolog | ND | 3 |
| Smad3 | Mothers against decapentaplegic, Drosophila Homolog of 3 | ND | ND |
| Smad7 | Mothers against decapentaplegic, Drosophila Homolog of 7 | ND | ND |
| Socs1 | Suppressor of citokyne signaling 1 | ND | 4 |
| Socs2 | Suppressor of citokyne signaling 2 | ND | ND |
| Stat1 | Signal Transducer and activator of transcription 1 | ND | ND |
| Stat3 | Signal Transducer and activator of transcription 3 | ND | ND |
| Stat4 | Signal Transducer and activator of transcription 4 | ND | ND |
| Stat6 | Signal Transducer and activator of transcription 6 | ND | ND |
| Tbx21 | T-Box 21 | ND | 10 |
| Tfrc | Transferrin receptor | ND | -5 |
| Tgfb1 | Transforming growth factor beta 1 | -2.2 | 2 |
| Tnf | Tumor necrosis factor | ND | 3.5 |
| Tnfrsf18 | Tumor necrosis factor receptor superfamily member 18 | ND | ND |
| Cd40 | CD40 antigen | ND | ND |
| Cd40lg | CD40 ligand | ND | ND |
| Fasl | Tumor Necrosis Factor Ligand Superfamily member 6 | 2 | ND |
| Vcam1 | Vascular cell adhesion molecule 1 | ND | ND |
| Vegfa | Vascular cellular adhesion molecule 1 | ND | 3.4 |
